# Preserved function and reduced angiogenesis potential of the quadriceps in patients with mild COPD

**DOI:** 10.1186/1465-9921-15-4

**Published:** 2014-01-17

**Authors:** Philippe Gagnon, Bruno B Lemire, Annie Dubé, Didier Saey, Alexandra Porlier, Marilie Croteau, Steeve Provencher, Richard Debigaré, François Maltais

**Affiliations:** 1 Centre de recherche, Institut Universitaire de cardiologie et de pneumologie de Québec, Université Laval, Québec, Canada

**Keywords:** Chronic obstructive pulmonary disease, Muscle, Muscle biopsy, Capillarization

## Abstract

**Background:**

Little is known about limb muscle abnormalities in mild COPD. Inactivity and systemic inflammation could play a role in the development of limb muscle dysfunction in COPD. The objective of the present study was to characterize quadriceps function, enzymatic activities and morphometry, levels of plasma inflammatory markers and physical activity levels in daily life (PA_dl_) in patients with mild COPD (GOLD 1).

**Methods:**

Mid-thigh muscle cross-sectional area (MTCSA), quadriceps strength, endurance, fiber-type distribution, capillarity, pro-angiogenesis factors (VEGF-A, angiopoietin I and II) and muscle oxidative capacity were assessed in 37 patients with mild COPD and 19 controls. Systemic inflammatory markers (CRP, IL-6, TNF-α, Fibrinogen, SP-D) and PA_dl_ were assessed.

**Results:**

MTCSA, quadriceps strength and endurance were not different between COPD and controls. Capillarity and muscle oxidative capacity were all preserved in mild COPD. Reduced pro-angiogenesis factor mRNA expression was seen in COPD. The level of moderately active intensity (>3 METs) was significantly lower in mild COPD and, in multiple regression analyses, the level of physical activity was a determinant of muscle oxidative capacity and capillarization. No between-group differences were found regarding muscle oxidative stress while circulating IL-6 levels were elevated in mild COPD.

**Conclusions:**

The quadriceps muscle function was preserved in mild COPD although a reduced potential for angiogenesis was found. The reduced level of daily activities and evidence of systemic inflammation in these individuals suggest that these factors precede the development of overt limb muscle dysfunction in COPD.

## Introduction

Chronic obstructive pulmonary disease (COPD) is often accompanied by important systemic consequences such as limb muscle dysfunction [1]. Limb muscle abnormalities in COPD include atrophy, weakness and increased fatigability [2-5]. At the morphological level, reduced proportion of oxidative fibers in conjunction with a relative increase in glycolytic fibers and reduced capillarity were also observed in the *vastus lateralis* of patients with moderate to severe COPD [6-8]. As a result of these morphological modifications, alterations of muscle metabolic profile, in favor of a predominant glycolytic metabolism, are seen during exercise in these patients [9,10]. The occurrence of limb muscle weakness and atrophy in COPD is not a trivial event because it contributes to exercise intolerance [11] and premature mortality [12] in this disease.

Why patients with COPD are vulnerable to the development of limb muscle functional and morphological disturbances is an important question to address if we wish to unravel the causes of this key systemic manifestation of the disease. Limb muscle dysfunction is likely to be multifactorial in nature, involving factors such as deconditioning, systemic inflammation and oxidative stress [13], hypoxemia [8] and nutritional imbalance.

Learning about the natural history of limb muscle dysfunction in COPD might provide useful insights about its underlying mechanisms of development. For example, if reduced physical activity and systemic inflammation were mechanistically linked to the development of limb muscle dysfunction, one would expect them to precede the development of limb muscle dysfunction. Studies reporting about limb muscle dysfunction in COPD mostly involve patients with advanced disease in whom inactivity, systemic inflammation and limb muscle dysfunction are well established making it impossible to know which one came first. In this investigation, we studied limb muscle function, enzymatic activities and morphometry in patients with mild COPD (GOLD 1) thinking that this would provide insights about the potential contribution of systemic inflammation, muscle oxidative stress and physical inactivity to the development of any muscle abnormalities. Our hypothesis was that systemic inflammation and physical inactivity would antedate overt evidence of limb muscle dysfunction in COPD and, as such, could be considered as mechanistically involved in this process.

Accordingly, we measured *i)* mid-thigh muscle cross sectional area (MTCSA), *ii)* quadriceps strength and endurance, fiber type proportion and surface, capillarity, oxidative capacity, pro-angiogenesis factors and oxidative stress, *iii)* levels of plasma inflammatory markers and i*v)* physical activity levels in daily life (PA_dl_) in patients with mild COPD as well as in age-matched healthy control subjects.

## Methods

See Additional file 1 for further information.

### Subjects

We studied 37 patients with GOLD 1 COPD [14] presenting a history of smoking (≥ 15 pack-years). Nineteen healthy age-matched subjects with normal spirometry and a history of smoking (≥ 15 pack-years) served as controls. None of them were involved in a previous study. Activity-related dyspnea was assessed by the *Baseline Dyspnea Index* (BDI) [15]. The research protocol was approved by the institutional ethics committee (Comité d’éthique, Institut Universitaire de cardiologie et de pneumologie de Québec, approval 20378) and all of the participants signed an informed consent prior to study enrolment. The sponsor (Boehringer Ingelheim and Pfizer) was not involved in the study design, data collection, analysis or interpretation. The sponsor had the opportunity to read and comment on the manuscript with no obligation for the authors to incorporate any suggestion into the final version.

### Study design

Participants were characterized during a first visit with pulmonary function tests, and measurements of body composition, MTCSA, quadriceps muscle strength and endurance. Comorbidities were assessed using a structured questionnaire and by medical chart review. During this initial visit, they also performed an incremental shuttle walking test. Following ≥ 72-hrs, but within a week of the first visit, a needle biopsy of the quadriceps was performed and blood was sampled to measure systemic inflammatory biomarkers. Finally, participants had to wear a physical activity monitor for 6 to 8 days.

### Pulmonary function testing

Standard pulmonary function tests, including lung volumes and diffusion capacity (DL_CO_) were obtained according to previously described guidelines [16] and related to predicted normal values [17]. Similarly, spirometry was performed according to guidelines [16] and related to predicted normal values [18].

### Body composition measurements

Body composition was measured at a standardized period of the day in a non-fasted state via direct bioelectrical impedance (*In Body 520, Biospace Co., Beverly Hills, CA*).

### Mid-thigh muscle cross-sectional area

Computed tomography was performed in the supine position and MTCSA was determined halfway between the pubic symphysis and the inferior surface of the femoral condyle.

### Quadriceps muscle strength and endurance

#### Strength measurements

Potentiated quadriceps twitch force (TwQ_pot_) of the dominant leg was measured by supramaximal magnetic stimulation of the femoral nerve 3 seconds following an isometric maximal voluntary contraction of the quadriceps (MVC) as previously reported [19].

#### Endurance protocol

Quadriceps endurance was evaluated according to an adapted version of the protocol previously reported by Allaire and colleagues [20]. It was defined as the time during which an isometric contraction at 50% of predetermined MVC could be maintained.

#### Incremental shuttle walking test (ISWT)

Peak oxygen uptake (VO2) was determined during a symptom-limited incremental shuttle walking test. The original protocol previously validated in COPD [21] was adapted to include 3 additional walking cadences in order to allow every participant to reach symptom limitation. During the test, subjects wore a facemask, linked to a portable gas exchange analyzer (*Oxycon Mobile, Viasys Healthcare, Jaeger, Germany*). Patients were allowed to run in order to attain maximal exercise capacity and standardized encouragements were provided to the patients.

### Muscle biopsy

Needle biopsies of the quadriceps were performed after 30-min of immobilization as described by Bergström [22] and as routinely done in our laboratory [10]. Muscle specimens were immediately frozen in liquid nitrogen and stored at -80°C for future analysis. From these muscle samples, fiber typing and surface areas were determined. The cross-sectional area of each fiber-type was calculated based on 40 randomly selected fibres of each type [23]. Muscle capillarity and pro-angiogenesis factors (vascular endothelial growth factor A [VEGF-A], angiopoietin I [Ang-I], angiopoietin II [Ang-II]) were measured. Finally, quadriceps oxidative (citrate synthase [CS], hydroxyacyl-coenzyme A dehydrogenase [HADH]) and glycolytic (phosphofructokinase [PFK]) enzymatic activity was assessed as well as muscle oxidative stress. See Additional file 1 for more details. [Detailed methods used to analyze fiber typing, muscle surface area, muscle capillarity, enzymatic activity, real-time PCR and oxidative stress].

### Systemic inflammation markers

Plasma levels of systemic inflammatory markers (TNF-α, IL-6, CRP, Fibrinogen, SP-D) were measured from antecubital venous blood sample.

### Levels of physical activity

Physical activity in daily life was monitored during 6 to 8 consecutive days via a portable device (*SenseWear® ArmBand, Bodymedia inc., Pittsburgh, USA*) worn on the right upper arm. Physical activity was further characterized by time and energy expenditure associated with at least moderate intensity (>3 METs). We report the mean daily values over the period of measure.

### Statistical analysis

All variables are expressed as means ± SD. Comparisons between the two groups for baseline characteristics, muscle functional and morphometric properties, level of oxidative stress and systemic inflammation were performed using unpaired *t*-test. The homogeneity of variances was analysed with the Brown-Forsythe test. Multiple regression analyses were performed to identify potential determinants of quadriceps MVC, oxidative capacity (CS activity) and capillary to fiber ratio. Independent variables that were used in this model included age, sex, smoking history (pack-years), fat-free mass index (FFMI), forced expiratory flow in 1 s (FEV_1_) % predicted, forced vital capacity (FVC) % predicted, total lung capacity (TLC) % predicted, functional residual capacity (FRC) % predicted, inspiratory capacity (IC) % predicted, residual volume (RV) % predicted, DL_CO_ % predicted, plasma IL-6 level, muscle oxidative stress indices, number of steps per day, energy expenditure > 3 METs and daily time > 3 METs. IL-6 was used in this model because it was the only inflammatory biomarker that was significantly increased in COPD. Beside this statistical consideration, IL-6 is the inflammatory biomarkers with the strongest association with important clinical outcomes such as mortality, in comparison with several other biomarkers, including TNF-α, CRP, fibrinogen and SP-D [24,25]. A statistical level of significance of 0.05 was used for all analysis. The data were analyzed using the statistical package program JMP (*Version 8.0.1, SAS Institute Inc., Cary, NC*).

## Results

### Subjects

Characteristics of the participants are presented in Table 1. Patients with mild COPD displayed more activity-related dyspnea in addition to mildly increased lung volumes and decreased diffusion capacity. Total smoking exposure tended to be lower in controls but this did not reach statistical significance. Peak VO_2_ was similar between the two groups. Comorbid conditions (hypertension, dyslipidemia, osteoporosis, anxiety) were similarly distributed between the two groups (Additional file 1) [Comorbidities for which participants were pharmacologically treated in patients with COPD and healthy controls].

**Table 1:** Subject characteristics

	**Controls (n =19)**	**Mild COPD (n =37)**
Age, yr	62 ± 8	65 ± 6
Male, %	69	68
BMI, kg/m^2^	27 ± 4	27 ± 4
Pack-years	36 ± 17	44 ± 21
BDI, (0–12)	10.6 ± 1.3	9.0 ± 1.6**
***Pulmonary function***		
FEV_1_, L	3.13 ± 0.70	2.76 ± 0.64*
% predicted	110 ± 19	96 ± 11**
FVC, L	3.91 ± 0.83	4.50 ± 0.98*
% predicted	104 ± 21	117 ± 15*
FEV_1_/FVC, %	81 ± 4	62 ± 6**
FEF_25-75%_, % predicted	101 ± 37	43 ± 14**
IC, % predicted	113 ± 16	114 ± 18
TLC, % predicted	102 ± 14	112 ± 11*
FRC, % predicted	94 ± 24	112 ± 23*
RV, % predicted	88 ± 19	107 ± 22*
DL_CO_, % predicted	97 ± 18	82 ± 19*
***Body composition***		
FFMI, kg/m^2^	19.1 ± 2.4	18.6 ± 2.3
FMI, kg/m^2^	8.6 ± 3.6	8.3 ± 2.6
***Functional capacity***		
$\dot{V}O_{2}$ _peak_, mL•kg^1^•min^-1^	27.5 ± 6.7	25.6 ± 4.8
$\dot{V}O_{2}$ _peak_, L•min^-1^	2.1 ± 0.6	1.9 ± 0.6
***Exercise limiting symptoms***		
Dyspnea, Borg score [0–10]	6.0 ± 2.0	6.9 ± 1.6
Leg fatigue, Borg score [0–10]	4.1 ± 1.7	5.3 ± 2.3

*Definitions of abbreviations:BMI* Body mass index, *BDI* Baseline dyspnea index, *FEV*_*1*_ forced expiratory volume in one second, *FVC* forced vital capacity, *FEF*_*25-75%*_ forced expiratory flow between 25 and 75% of FVC, *IC* inspiratory capacity, *TLC* total lung capacity, *FRC* functional residual capacity, *RV* residual volume, *DL*_*CO*_ diffusion capacity, *FFMI* Fat-free mass index, *FMI* Fat mass index, $\dot{V}O_{2}$ oxygen uptake. Values are mean ± SD. *p < 0.05 vs. healthy controls; **p < 0.0001 vs. healthy controls.

### Quadriceps cross-sectional area and function

**Figure 1:**
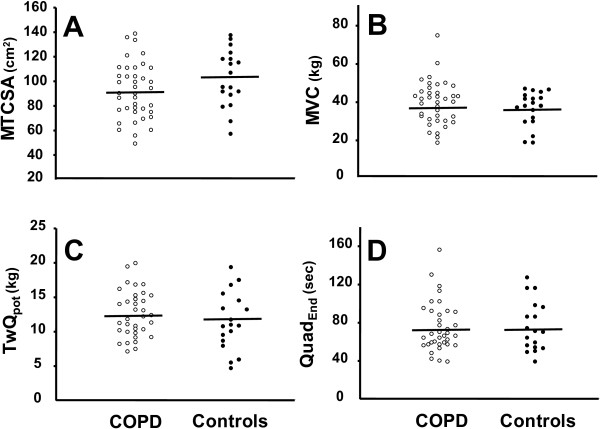
**Individual values for mid-thigh cross-sectional area (MTCSA, *panel A*), maximal voluntary contraction of the quadriceps (MVC, *panel B*), mean potentiated quadriceps twitch force at baseline (TwQ**_**pot**_**, *panel C*), time to exhaustion during an isometric contraction at 50% of MVC (Quad**_**End**_**, *panel D*) in patients with mild COPD (*open dots*) and controls (*filled dots*).** The horizontal lines represent group mean values.

No difference was found in MTCSA between patients with mild COPD and healthy controls (Figure 1A). Likewise, MVC, TwQ_pot_ and endurance were all similar between patients with mild COPD and controls (Figure 1B-D).

### Quadriceps intrinsic characteristics

#### Fiber typing and surface areas

The fiber type distribution was similar between controls and patients with COPD, showing a slight type I fiber predominance over type II for both groups (Table 2). The respective mean muscle surface area occupied by type I and II fibers were not different between mild COPD and controls.

**Table 2:** Quadriceps muscle characteristics

	**Controls**	**Mild COPD**	**p-value**
***Fiber type distribution***			
Type I fibers, % total fibers	57 ± 14	55 ± 13	0.59
Type II fibers, % total fibers	43 ± 14	45 ± 13	0.59
***Fiber type area***			
Type I fibers, μm^2^	3403 ± 2691	2486 ± 3416	0.33
Type II fibers, μm^2^	2635 ± 1868	1919 ± 2683	0.32
Total, μm^2^	6038 ± 4503	4405 ± 6079	0.32
***Muscle capillarity***			
Type I fibers, capillary per fiber	3.80 ± 1.24	3.33 ± 1.04	0.15
Type II fibers, capillary per fiber	2.90 ± 1.04	2.63 ± 0.90	0.35
Total, capillary per fiber	3.38 ±1.09	2.92 ± 0.87	0.10
***Muscle capillarity to fiber area ratio***			
Type I fibers, capillary per fiber μm^2^	1.46 ± 1.56	2.00 ± 1.16	0.16
Type II fibers, capillary per fiber μm^2^	1.70 ± 1.56	2.51 ± 1.51	0.08
Total, capillary per fiber μm^2^	1.91 ± 1.78	2.35 ± 1.24	0.30
***Muscle enzymatic activity***			
*Oxidative profile*			
HADH, μmol•min^-1^•g muscle^-1^	2.98 ± 2.56	3.83 ± 4.35	0.47
CS, μmol•min^-1^•g muscle^-1^	11.86 ± 6.10	14.49 ± 3.66*	0.04
*Glycolytic profile*			
PFK, μmol•min^-1^•g muscle^-1^	34.59 ± 9.04	31.98 ± 8.32	0.29
***Muscle oxidative stress***			
4-HNE, AU	0.99 ± 0.35	1.24 ± 0.67	0.14
OxyBlot®, AU	0.74 ±0.57	1.22 ± 1.46	0.19

*Definitions of abbreviations:HADH* hydroxyacyl-coenzyme A dehydrogenase, *CS* citrate synthase, *PFK* phosphofructokinase, *4-HNE* 4-hydroxynonenal, *AU* arbitrary units. Values are mean ± SD. *p < 0.05 vs. healthy controls.

#### Muscle capillarity and angiogenesis

The number of capillaries in contact with type I and type II muscle fibers were not different between groups (Table 2). A similar conclusion was reached when taking into account the surface area of type I and II fibers.

**Figure 2:**
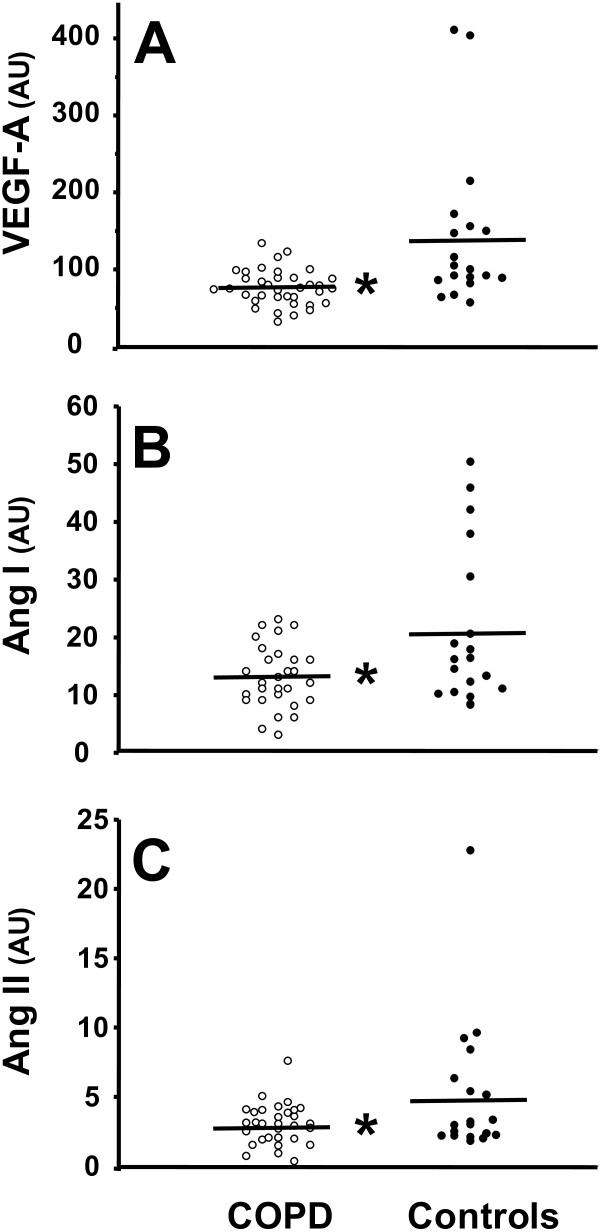
**Individual values of quadriceps mRNA expression for vascular endothelial growth factor A (VEGF-A, panel A), angiopoietin I (Ang I, panel B) and angiopoietin II (Ang II, panel C) in patients with mild COPD (*open dots*) and controls (*filled dots*).** The horizontal lines represent group mean values. *Indicates statistically significant difference between the two groups (see text for exact p values).

The mRNA expressions of the pro-angiogenesis factors are provided in Figure 2. When compared with healthy controls, VEGF-A (p < 0.001), angiopoietin I (p = 0.002) and II (p = 0.01) mRNA expression levels were lower in patients with mild COPD.

#### Enzymatic activity

The activity of CS was significantly higher in patients with mild COPD while the activity of HADH (oxidative) and PFK (glycolytic) were similar between groups (Table 2).

#### Oxidative stress

We found no differences between patients with mild COPD and controls for the muscle oxidative stress (Table 2).

### Systemic inflammation

Plasmatic levels of IL-6 were significantly higher in patients with COPD and CRP levels tended to be higher (p = 0.08) when compared with healthy controls. On the opposite, plasmatic levels of fibrinogen were higher in healthy controls (Table 3).

**Table 3:** Levels of plasma inflammatory markers

	**Controls**	**Mild COPD**	**p-value**
***Systemic inflammation***			
CRP, mg•L^-1^	1.44 ± 1.36	2.79 ± 3.22	0.08
IL-6, pg•L^-1^	3.37 ± 2.19	5.04 ± 2.88*	0.03
TNF-α, pg•mL^-1^	2.37 ± 3.12	2.46 ± 1.53	0.89
Fibrinogen, mg•mL^-1^	3.45 ± 2.49	2.34 ± 0.63*	0.01
SP-D, ng•mL^-1^	12.12 ± 7.49	15.83 ± 14.62	0.30

*Definitions of abbreviations:CRP* C-reactive protein, *IL-6* Interleukin-6, *TNF-α* Tumor necrosis factor-α, *SP-D* surfactant protein D. Values are mean ± SD. *p < 0.05 vs. healthy controls.

### Levels of physical activity

**Figure 3:**
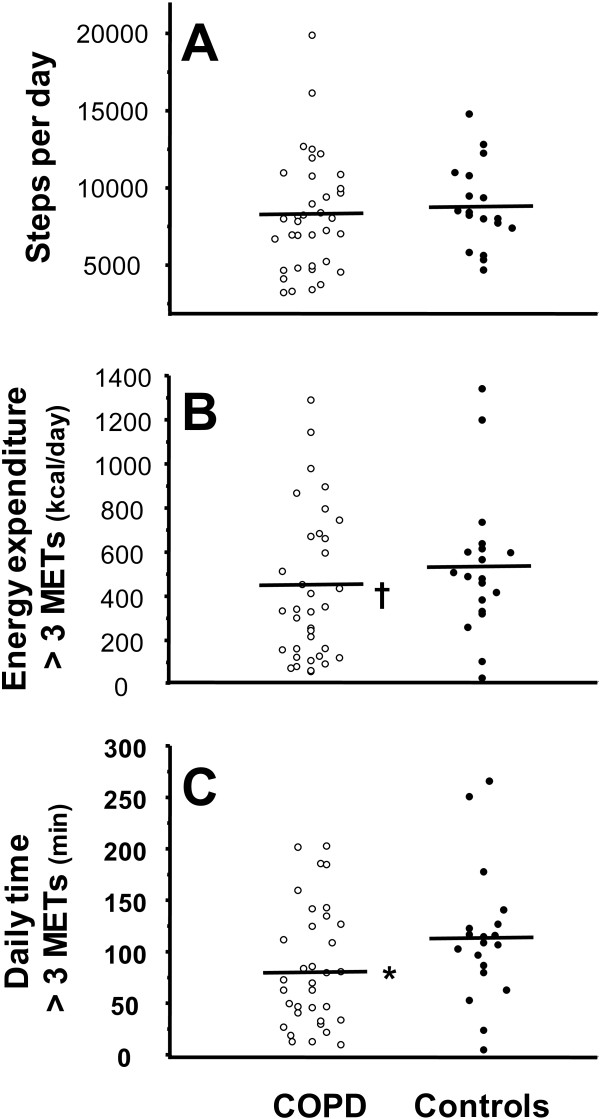
**Individual values for mean daily steps (*panel A*), mean daily energy expenditure of at least moderate intensity (> 3 metabolic equivalent (METs)) (*panel B*) and mean daily time spent > 3 METs (p*anel C*) in patients with mild COPD (*open dots*) and controls (*filled dots*).** The horizontal lines represent group mean values. *p < 0.05 vs. controls; ^†^p = 0.09 vs. controls.

Levels of physical activities in daily life are reported in Figure 3. Patients with COPD had similar daily step count when compared with healthy controls. However, when accounting for the intensity of daily life activities, patients with mild COPD showed a decreased amount of at least moderate intensity (> 3 METs) physical activities in daily life. Accordingly, mean daily time dedicated to at least moderate intensity daily activities was significantly reduced in patients with mild COPD.

### Correlates of quadriceps muscle function

In multiple regression analysis, quadriceps MVC was positively associated with male gender (p = 0.03) and FFMI (p = 0.003) and negatively with FRC (p = 0.002). Altogether the model explained 73% of the variance in MVC. CS activity was positively associated with energy expenditure > 3 METs (p < 0.001) and daily time > 3 METs (p = 0.007) with a total r^2^ = 0.53. The capillary-to-fiber ratio was positively associated with number of steps per day (p = 0.045), energy expenditure > 3 METs (p < 0.001) and daily time > 3 METs (p = 0.007) with a total r^2^ = 0.49. Plasma IL-6 levels and indices of muscle oxidative stress did not emerge as significant determinants of quadriceps MVC, oxidative capacity and capillarization.

## Discussion

This study is the first to directly evaluate both, functional and intrinsic muscle properties exclusively in patients with mild (GOLD 1) COPD. The main finding of this study was that muscle mass and function, as well as morphometric and enzymatic properties were generally preserved in patients with mild COPD when compared with healthy controls of similar age and smoking history. Despite this, subtle muscle abnormalities were already present in GOLD 1 patients with reduced expression of pro-angiogenesis factors. The findings that the level of physical activity of at least moderate intensity was already decreased in these patients and its correlation with muscle MVC and capillarity support a role for reduced physical activity in the initiation of limb muscle dysfunction in COPD.

### Quadriceps function and morphometry

Reduced muscle strength and endurance were previously reported in patients with GOLD 1 and 2 COPD [26]. One asset of the present study is that only patients with GOLD 1 COPD were enrolled, allowing to conclude about the preservation of muscle mass, strength and endurance in this specific patient population.

Our data suggests that fiber type shifting and metabolic alterations are not early events in the process leading to limb muscle dysfunction in COPD. This finding is consistent with the fact that the fiber type shifting of the quadriceps is associated with COPD severity in patients with GOLD 3 and 4 COPD [27,28]. Furthermore, the proportion of type I fibers in our patients was well beyond the proposed pathological threshold of 27% [27], highlighting that muscle fiber type shifting occurs in more advanced disease. Lastly, evidence of reduced oxidative enzyme activities were not found in another study involving patients with mild to moderate COPD [29].

Our results about the preservation of muscle capillarization in mild COPD are consistent with a recent study reporting a similar number of capillaries in contact with type I and II muscle fibers between patients with mild COPD and healthy controls in the *tibialis anterior*[30]. Despite this, a decreased mRNA expression level of pro-angiogenesis factors was found in COPD. The reduced quadriceps VEGF-A mRNA expression is consistent with a previous report [31]. In contrast to our findings, elevated angiopoietin II mRNA expression level was previously reported in the quadriceps of patients with severe COPD [32]. A negative association between angiopoietin II mRNA expression and FEV_1_ was also observed in this study [32] suggesting that the divergent conclusions reached about the quadriceps angiopoietin II expression could well be explained by differences in disease severity between study populations. Muscle angiogenesis is regulated by oxidative stress and inflammation [32], two features that are more likely to predominate in advanced COPD or during disease exacerbation. As such, the angiogenesis status of the quadriceps may differ according to disease severity.

Skeletal muscle angiogenesis is a highly regulated process involving several coordinated signaling pathways, including VEGF and the angiopoietin system whose activation precede and promote the formation of new capillaries [33]. Considering the influence of exercise on these key angiogenesis regulating pathways, reduced physical activity level emerges at a likely explanation for the reduction in angiogenic regulating pathways in patients with GOLD 1 COPD. In the absence of decreased capillarization, we could only speculate on the significance of the reduced pro-angiogenesis factors. One possibility is that biochemical regulation of the angiogenic process precedes the observable changes in capillarization [34]. This interpretation implies that patients with COPD could be vulnerable in situation where an increase capillarization would be required such as during hypoxemia. Alternatively, it can be submitted that muscle capillarization is fairly stable and relatively insensitive to the downregulation in angiogenic growth factors at this stage of the disease [35].

### Mechanisms of limb muscle dysfunction in mild COPD

Similarly to other investigators [31,36-38], we found only equivoqual evidence of systemic inflammation in this population of mild COPD. Overall, our inflammatory biomarker data is consistent with the available literature in showing that IL-6 elevation is very consistent in COPD [38]. Our findings are also congruent with a previous study showing that TNF-α and surfactant-D protein (SP-D) levels are not increased in GOLD 2–4 COPD compared to smokers with normal lung function while fibrinogen and CRP levels are highly variable in COPD [38]. The finding of a reduced plasma fibrinogen level in GOLD 1 COPD was unexpected. Fibrinogen elevation was reported in a subset of the ECLIPSE cohort involving patients with GOLD 2–4 COPD, in comparison smokers with normal lung function [38]. However, significant overlap between COPD and controls exists in such a way most patients with COPD have normal fibrinogen levels [37]. Another point to consider in interpreting this finding is that our investigation involved patients with GOPD 1 COPD, for whom little information exists about the inflammatory status. Lastly, the relative small sample size of our investigation should be considered in interpreting the inflammatory biomarker data.

In the context that muscle oxidative stress was not present in patients with GOLD 1 COPD and because plasma IL-6 and indices of muscle oxidative stress were not associated with muscle function and capillarization, our results do not support the thesis that systemic inflammation or muscle oxidative stress initiate the establishment of limb muscle dysfunction associated with COPD.

Shrikrishna and colleagues reported reduced *rectus femoris* surface area and physical activity level in patients with GOLD 1 COPD in comparison to healthy controls [39]. Interestingly, and in contrast to the present study, the smoking exposure of their healthy controls was much lower than in COPD. This may suggest that smoking by itself may be associated with reduced muscle mass as previously suggested [40]. Altogether, these studies would support the idea that smoking exposure is important in the early development of limb muscle atrophy in COPD.

Our results provide further confirmation that the level of moderately intense physical activity is already low in GOLD I COPD [39,41,42]. The correlative findings between the level of moderately intense physical activity and quadriceps MVC and capillarity support a role for reduced physical activity in the initiation of limb muscle dysfunction in COPD. This is in line with recent evidences also pointing to the potential role of reduced physical activity level in the development of quadriceps weakness in COPD [43].

### Methodological considerations

Although our results are based on a relatively limited sample size, they exclusively pertain to patients with mild (GOLD 1) COPD. Subjects were extensively characterized and the muscle profile was well defined. In fact, the levels of physical activity could be related to intrinsic and functional muscle properties, offering a unique opportunity to speculate on causal relationship in locomotors muscles alterations in early COPD. However, a definitive answer to this issue could only be answered by longitudinal studies evaluating the interactions between physical activity and limb muscle function over time.

### Clinical perspectives

Exercise training remains the most relevant strategy to reverse functional and metabolic impairments occurring in the skeletal muscle: it is known to partially reverse the loss of muscle cross-sectional area and improve muscle capillary density [44], irrespective of the disease severity when considering GOLD 1 to 4 patients [45]. Accordingly, our results provide support for the use of pulmonary rehabilitation in patients with GOLD 1 COPD as they already show subtle muscle abnormalities and reduced level of physical activity.

In conclusion the present study indicates that limb muscle function, fiber type distribution, enzymatic activities and capillarization was generally preserved in mild (GOLD 1) COPD. The level of muscle pro-angiogenesis factors was nevertheless reduced in these patients suggesting that pre-clinical muscle abnormalities were already present. Our findings also support a role for reduced physical activity in the initiation of limb muscle dysfunction in COPD.

## Competing interests

The authors have no competing interests to declare about this study.

## Authors’ contributions

All authors were substantially involved in design, acquisition, analysis and interpretation of the study. All authors contributed to the intellectual content of the manuscript and were consulted for final approval of the submitted version. Accordingly, we did not omit to include any other author that would fulfill these authorship requirements. PG, BBL, AD, DS, AP, MC, SP, RD, FM. Conception and design: PG, BBL, DS, SP, RD FM. Acquisition of data: PG, BBL, AD, AP, MC, FM. Analysis and interpretation: PG, BBL, AD, DS, SP, RD, FM. Drafting the article for important intellectual content: PG, BBL, AD, DS, SP, RD, FM. Final approval of the version to be submitted: PG, BBL, AD, DS, AP, MC, SP, RD, FM. All authors read and approved the final manuscript.

## Supplementary Material

Additional file 1 Preserved function and reduced angiogenesis potential of the quadriceps in patients with mild COPD: supplementary data. Click here for file
