# Supplementary material for: Preserved function and reduced angiogenesis potential of the quadriceps in patients with mild COPD
Source: Respir Res. 2014 Jan 17;15(1):4. doi: 10.1186/1465-9921-15-4 (PMC3898018; doi:10.1186/1465-9921-15-4)
Supplement: Additional file 1 — Preserved function and reduced angiogenesis potential of the quadriceps in patients with mild COPD: supplementary data. [file 1465-9921-15-4-S1.docx]

**Online material**

**PRESERVED FUNCTION AND REDUCED ANGIOGENESIS POTENTIAL OF THE QUADRICEPS IN PATIENTS WITH MILD COPD**

**Authors:**

Philippe GAGNON, PhD^1^, philippe.gagnon@criucpq.ulaval.ca

Bruno LEMIRE, PhD^1^, bruno.lemire@criucpq.ulaval.ca

Annie DUBÉ, PhD^1^, annie.dube@criucpq.ulaval.ca

Didier SAEY, PhD^1^, [didier.saey@rea.ulaval.ca](mailto:didier.saey@rea.ulaval.ca)

Alexandra PORLIER, BSc^1^, [alexandra.porlier@criucpq.ulaval.ca](mailto:alexandra.porlier@criucpq.ulaval.ca)

Marilie CROTEAU, BSc^1^, marilie.croteau@criucpq.ulaval.ca

Steeve PROVENCHER, MD, MSc^1^, steve.provencher@cricupq.ulaval.ca

Richard Debigaré, PhD^1^, richard.debigare@rea.ulaval.ca

François MALTAIS, MD^1^, francois.maltais@med.ulaval.ca

**Affiliation :**

^1^Centre de recherche, Institut Universitaire de cardiologie et de pneumologie de Québec, Université Laval, Québec, Canada.

**Address of correspondence:** Dr François Maltais

Institut Universitaire de cardiologie et de

pneumologie de Québec

2725 Chemin Ste-Foy

Québec, Québec

G1V 4G5

CANADA Tel: 418-656-4747; Fax: 418-656-4762

E-mail: francois.maltais@med.ulaval.ca

**METHODS**

## Subjects

We studied 37 patients with GOLD I COPD (post-bronchodilator forced expiratory volume in 1 second [FEV_1_] > 80% predicted and FEV_1_/forced vital capacity [FVC] < 0.7) [[1](#_ENREF_1)] presenting a history of smoking (≥ 15 pack-years). Thirteen healthy age-matched subjects with normal spirometry (FEV_1_ >80% predicted and FEV_1_/FVC ≥0.7) and a history of smoking (>10 pack-years) served as controls.

Participants were recruited following their recent participation in COPD or lung cancer screening studies (n = 30) or following consultation with a chest physician (n = 7). Subjects were excluded if they presented any medical condition, other than COPD, likely to influence muscle condition (i.e. cardiovascular, neurological, musculoskeletal, locomotor or other respiratory diseases as well as ß-blockers therapy). Subjects were asked to avoid alcohol, caffeine and heavy meals 3 hours before the visit and high intensity physical activity for at least 24 hours before testing. Experimental visits were conducted at the same time of the day for every subject.

**Pulmonary function testing**

The predicted value for inspiratory capacity (IC) was obtained by subtracting the functional residual capacity (FRC) predicted value from total lung capacity (TLC) predicted value. Reported values of baseline spirometry in the first visit were measured after the inhalation of 400 µg of salbutamol in every participant [[1](#_ENREF_1)].

**Mid-thigh cross-sectional area**

Computed tomography was performed in the supine position (*Siemens Sensation 64, Erlanger, Germany*) and MTCSA was determined halfway between the pubic symphysis and the inferior surface of the femoral condyle. MTCSA was calculated using normal-density areas with attenuation between 35 and 100 Hounsfield units (HU) [[2](#_ENREF_2)].

### Quadriceps muscle strength and endurance

### Strength measurements. Each maximal voluntary contraction (MVC) maneuver was separated by 30 seconds and verbal encouragements were provided during the maneuver. Superimposed twitch were assessed during the MVC maneuvers to ensure voluntary contractions were maximal. MVC maneuvers were excluded from further analysis when twitch superimposition produced additional forces. The reported values correspond to the mean of the three strongest contractions for potentiated quadriceps twitch force (TwQ_pot_).

**Endurance protocol.** Quadriceps endurance was evaluated according to an adapted version of the protocol previously reported by Allaire and colleagues [[3](#_ENREF_3)]. It was defined as the time during which an isometric contraction at 50% of predetermined maximum voluntary contraction (MVC) could be maintained. Briefly, after 10 minutes of resting in the sitting position, patients were asked to maintain an isometric contraction of the quadriceps at 50% of their predetermined MVC until exhaustion. A visual feedback was used to help subjects maintain the pre-determined tension. The quadriceps muscle endurance represented the time to exhaustion, defined as the time at which the tension was less than 40 % MVC.

### Muscle biopsy

**Fiber typing and surface areas.** Briefly, 5 μm cryosections were prepared from frozen muscle samples. Muscle was immunohistochemically stained using monoclonal anti-skeletal myosin fast (*Sigma, Oakville, ON, Canada*) at 4^o^C with 1:200 dilution and VECTASTAIN Elite ABC system (*Vector Laboratories, Burlington, ON, Canada*). Antibody localizes an epitope on the myosin heavy chain and stains the fast (type II) and neonatal isomyosin molecules found in skeletal muscle. Muscle fibers were classified according to the staining intensity: type I (non-stained) and type II (stained). All fibers were counted with an image analysing system (*Image Pro Plus 4.5 for Windows, MediaCybernetics, Silver Spring, MD, USA*) and classified to obtain the fiber-type composition for each subject. The proportion of types I (non-stained) and II (stained) fibers was assessed and calculated as the number of fibers of each type divided by the total number of muscle fibers. We used the technique previously developed in our laboratory to assess muscle fiber area [[4](#_ENREF_4)]. Accordingly, muscle sections were magnified and transmitted to an image analyzer (*Image Pro Plus 4.5 for Windows, MediaCybernetics, Silver Spring, MD, USA*) to count and classify fibers and determine their mean CSA, which was calculated based on 40 randomly selected fibers of each type.

**Muscle capillarity.** Briefly, 5 μm cryosections were prepared from frozen muscle samples. Muscle was immunohistochemically stained using monoclonal mouse anti-human CD31 endothelial cell (*Clone JC70OA : Dako, Burlington, ON, Canada*) 1:20 dilution and EnVision + System-HRP (AEC) kit (*Dako, Burlington, ON, Canada*). Capillaries were counted with an image analysing system (*Image Pro Plus 4.5 for Windows, MediaCybernetics, Silver Spring, MD, USA*). The mean capillary contact/fibre-type ratio was obtained by averaging, on 40 randomly selected fibres of each type, the number of capillaries in direct contact with their outer membrane.

**Enzymatic activity.** Quadriceps oxidative (citrate synthase [CS] and hydroxyacyl-coenzyme A dehydrogenase [HADH]) and glycolytic (phosphofructokinase [PFK]) enzymatic activity were assessed using spectrophotometric techniques as previously described [[5](#_ENREF_5), [6](#_ENREF_6)]. The reaction media used to measure enzymatic activities were *i*) tris-HCl 150 mM (pH 8.0) containing DTNB 0.16 mM, acetyl-CoA 1.235 M, and sodium oxaloacetate 0.38 mM for CS *ii*) triethanolamine 100 mM (pH 7.0) containing EDTA 5 mM, NADH 0.17 mM, KCN 1 mM and acetyl-CoA 0.1 mM for HADH and *iii*) and tris-HCl 50 mM (pH 8.2) containing MgCl_2_ 6 mM, KCl 250 mM, KCN 1 mM, ATP 1 mM, AMP 1 mM, NADH 0.17 mM fructose-6-phosphate 3 mM, glycerol-3-phosphate deshudrogenase 2 U, aldolase 0.18 U, triose phosphate isomerase 1.92 U for PFK. Each assay was done in duplicate and the average of the two values is reported.

**Real-Time PCR.** Angiogenic factors (Vascular endothelial growth factor A (VEGF-A), Angiopoietin I (Ang-I), Angiopoietin II (Ang-II)) were characterized using messenger ribonucleic acid (mRNA) expression by real-time PCR. Total RNA extraction was performed from ≈ 15 mg of muscle using a commercially available preparation (*TRIzol^®^ Reagent, Invitrogen, Burlington, ON, Canada*). One μg of RNA was reverse transcribed to cDNA using Quantitect^TM^ Reverse Transcription Kit (*Qiagen, Mississauga, ON, Canada*). Real-time PCR were performed in a Rotor-Gene^TM^ 6000 (*Corbett Life Science, San Francisco, CA, USA*) using Quantitect^TM^ SYBR^®^ Green PCR Kit (*Qiagen, Mississauga, ON, Canada*). All protocols consisted of one denaturing cycle at 90°C for 3 minutes, followed by 40 cycles of denaturing at 90°C for 30 seconds, annealing at 60°C for 30 seconds and elongation at 72°C for 60 seconds followed by final elongation at 72°C for 5 minutes. At the end of the PCR amplifications the samples were subjected to a melting curve analysis. The comparative threshold cycles (^∆^CT) values were normalized for RPLPO human reference gene and analyzed using the 2^-∆Ct^ method [[7](#_ENREF_7)]. All RT-PCR runs were performed in duplicate to ensure quantitative accuracy. Finally, the primer sequence used for Real-time PCR analyses are shown in Table E1.

**Oxidative stress.** Total protein extraction was performed with ≈ 30 mg of muscle. Muscle samples were then homogenised using a Polytron PowerGen 125 (*Omni International, Marietta, GA, USA*). Protein content was determined by a DC protein essay (*BioRad, Mississauga, ON, Canada*) based on a modified Lowry method. For lipid peroxidation measurements, electrophoresis was performed using the primary antibody 4-HNE (*Calbiochem, San Diego, CA, USA cat. no. 393207)* on a 12 % SDS PAGE gels. After a 2-hour protein transfer in cold (4°C) buffer, nitrocellulose membranes were blocked for 1 hour with 5% non-fat dry milk in TBS-T (Tris 10 mM pH 8, NaCl 150 mM containing 0,1% Tween-20). The membranes were then incubated overnight at 4°C using the primary antibody 4-HNE (*Calbiochem, San Diego, CA, USA cat. no. 393207*). The membranes were then washed 3 x 10 minutes with TBS-T and incubated for 1 hour at room temperature with the secondary antibody IRDye 800CW goat anti-rabbit IgG (H+L) (*Mandel, Guelph, ON, Canada cat. no. LIC-926-32211*) and again washed as above.

To assess protein carbonylation, the OxyBlot® kit (*Millipore, Billeria, MA, USA*) was used to detect carbonyl groups introduced into proteins by oxidative reactions with oxides of nitrogen and was done according to the manufacturer’s protocol, except for the secondary antibody. After a 2-hour protein transfer in cold (4°C) buffer, nitrocellulose membranes were blocked for 1 hour with BSA 1% in TBS-T. The membranes were then incubated 1 hour at room temperature using the primary antibody provided in the OxyBlot® kit. The membranes were then washed 3 x 10 minutes with TBS-T and incubated for 1 hour at room temperature with the secondary antibody IRDye 800CW goat anti-rabbit IgG (H+L) and again washed as above. All protein contents were normalized to α-tubulin (*Sigma, Oakville, ON, Canada cat. no. T5168*) with secondary antibody IRDye 800CW goat anti-mouse IgG (H+L) (*Mandel, Guelph, ON, Canada cat. no. LIC-926-32220*). Quantification was performed using infrared imaging system (*Odyssey, LI-COR: Mandel, Guelp, ON, Canada*). Representative blots that were used to assess lipid peroxidation and protein carbonylation are provided in Figure E1.

**Systemic inflammation markers**

The antecubital venous blood was sampled after 10-min of rest and centrifuged for 10-min. Plasma was aliquoted, and stored at -80°C until further analysis. Commercial ELISA kits *(R&D Systems Inc, Minneapolis, MN, USA and Genway Biotech, San Diego, CA, USA)* were used to measure the plasma levels of systemic inflammatory markers (TNF-α, IL-6, CRP, Fibrinogen, SP-D).

**RESULTS**

## Subjects

The comorbidities for which our participants were pharmacologically treated during the study were hypertension (CTRL, n=4; COPD, n=11), osteoporosis (CTRL, n=2; COPD, n=8), hypercholesterolemia (CTRL, n=3; COPD, n=12) and depression/anxiety (CTRL, n=3; COPD, n=2). Finally, two mild COPD patients were treated with inhaled corticosteroid on a regular base.

**Levels of physical activity**

The portable monitor used to assess levels of physical activity in daily life was worn for a balanced number of days in both groups (6.8 ± 0.8 vs. 6.8 ± 0.5 days) for CTRL and COPD respectively). Both groups also demonstrated good compliance with the monitor as they both wore it for a similar daily period of time (14.2 ± 1.9 vs. 13.7 ± 1.5 hours/day for CTRL and COPD respectively).

**Correlates of quadriceps muscle function**

In multiple regression analysis, quadriceps MVC was positively associated with male gender (p = 0.03) and FFMI (p = 0.003) and negatively with FRC (p = 0.002). Altogether the model explained 73% of the variance in MVC. The partial R related to each parameter included in the model to explain MVC variance in our stepwise regression analysis is presented in Table E2. As can be seen, the most important contributor to MVC was fat-free mass index.

**REFERENCES**

1. Vestbo J, Hurd SS, Agusti AG, Jones PW, Vogelmeier C, Anzueto A, Barnes PJ, Fabbri LM, Martinez FJ, Nishimura M, et al: **Global strategy for the diagnosis, management, and prevention of chronic obstructive pulmonary disease: GOLD executive summary.** *Am J Respir Crit Care Med* 2013, **187:**347-365.

2. Kelley DE, Slasky BS, Janosky J: **Skeletal muscle density: effects of obesity and non-insulin-dependent diabetes mellitus.** *Am J Clin Nutr* 1991, **54:**509-515.

3. Allaire J, Maltais F, Doyon JF, Noël M, Leblanc P, Carrier G, Simard C, Jobin J: **Peripheral muscle endurance and the oxidative profile of the quadriceps in patients with COPD.** *Thorax* 2004, **59:**673-678.

4. Whittom F, Jobin J, Simard PM, Leblanc P, Simard C, Bernard S, Belleau R, Maltais F: **Histochemical and morphological characteristics of the vastus lateralis muscle in patients with chronic obstructive pulmonary disease.** *Med Sci Sports Exerc* 1998, **30:**1467-1474.

5. Maltais F, Leblanc P, Whittom F, Simard C, Marquis K, Bélanger M, Breton MJ, Jobin J: **Oxidative enzyme activities of the vastus lateralis muscle and the functional status in patients with COPD.** *Thorax* 2000, **55:**848-853.

6. Zammit VA, Newsholme EA: **The maximum activities of hexokinase, phosphorylase, phosphofructokinase, glycerol phosphate dehydrogenases, lactate dehydrogenase, octopine dehydrogenase, phosphoenolpyruvate carboxykinase, nucleoside diphosphatekinase, glutamate-oxaloacetate transaminase and arginine kinase in relation to carbohydrate utilization in muscles from marine invertebrates.** *Biochem J* 1976, **160:**447-462.

7. Livak KJ, Schmittgen TD: **Analysis of relative gene expression data using real-time quantitative PCR and the 2(-Delta Delta C(T)) Method.** *Methods* 2001, **25:**402-408.

**FIGURE LEGENDS**

**FIGURE E1.** Lipid peroxidation (4-HNE, panel A) was assessed using standard immunoblot techniques where 4-HNE (in green) was expressed relative to tubulin (in red), each gel containing 4 subjects. Subjects (represented as number) were placed as monoplica onto gels, but 2 independent gels were made for all subjects. Protein carbonylation was quantified with immunoblotting (OxyBlot®, panel B). Each gel contained 2 subjects whose sample was analyzed in duplicate to quantify oxidized and non-oxidized protein levels (negative control).

**TABLE E1.**

**Primer sequence used for RT-PCR analyses**

| Target PCR primer sequence 5’→ 3’  mRNA  VEGF-A Forward GCAGCTTGAGTTAAACGAACG  Reverse GGTTCCCGAAACCCTGAG  Ang-I Forward CCTGATCTTACACGGTGCTGATT  Reverse GTCCCGCAGTATAGAACATTCCA  Ang-II Forward ATAAGCAGCATCAGCCAACCA  Reverse CATTCCGTTCAAGTTGGAAGGA  RPLPO Forward TCTACAACCCTGAAGTGCTTGATATC  Reverse GCAGACAGACACTGGCAACATT |  |
| --- | --- |

**TABLE E2.**

**Results of the stepwise regression analysis for maximal voluntary contraction (MVC)**

|  | *Partial R^2^* | *Cumulative R^2^* | *P value* |
| --- | --- | --- | --- |
| *FFMI (kg/m^2^)* | *0.78* | *0.61* | *0.001* |
| *FRC (% predicted)* | *-0.30* | *0.70* | *0.002* |
| *Sex (M/F)* | *0.17* | *0.73* | *0.03* |

Definitions of abbreviations: FFMI = Fat-free mass index; FRC = functional residual volume.
